# CRISPR/Cas9–Mediated Genome Editing for *Pseudomonas fulva*, a Novel *Pseudomonas* Species with Clinical, Animal, and Plant–Associated Isolates

**DOI:** 10.3390/ijms23105443

**Published:** 2022-05-13

**Authors:** Nan Zhang, Jintao He, Abrar Muhammad, Yongqi Shao

**Affiliations:** 1 Max Planck Partner Group, Faculty of Agriculture, Life and Environmental Sciences, Institute of Sericulture and Apiculture, College of Animal Sciences, Zhejiang University, Hangzhou 310058, Chinaabrar_ento334@zju.edu.cn (A.M.); 2 Key Laboratory for Molecular Animal Nutrition, Ministry of Education, Hangzhou 310058, China

**Keywords:** *Pseudomonas fulva*, CRISPR–Cas9, gene editing, antibiotic resistance, *gfp*

## Abstract

As one of the most widespread groups of Gram–negative bacteria, *Pseudomonas* bacteria are prevalent in almost all natural environments, where they have developed intimate associations with plants and animals. *Pseudomonas fulva* is a novel species of *Pseudomonas* with clinical, animal, and plant–associated isolates, closely related to human and animal health, plant growth, and bioremediation. Although genetic manipulations have been proven as powerful tools for understanding bacterial biological and biochemical characteristics and the evolutionary origins, native isolates are often difficult to genetically manipulate, thereby making it a time–consuming and laborious endeavor. Here, by using the CRISPR–Cas system, a versatile gene–editing tool with a two–plasmid strategy was developed for a native *P. fulva* strain isolated from the model organism silkworm (*Bombyx mori*) gut. We harmonized and detailed the experimental setup and clarified the optimal conditions for bacteria transformation, competent cell preparation, and higher editing efficiency. Furthermore, we provided some case studies, testing and validating this approach. An antibiotic–related gene, *oqxB*, was knocked out, resulting in the slow growth of the *P. fulva* deletion mutant in LB containing chloramphenicol. Fusion constructs with knocked–in *gfp* exhibited intense fluorescence. Altogether, the successful construction and application of new genetic editing approaches gave us more powerful tools to investigate the functionalities of the novel *Pseudomonas* species.

## 1. Introduction

The genus *Pseudomonas* encompasses the most diverse and ecologically significant group of Gram–negative bacteria in nature. Members of the genus are ubiquitous and many of them have developed intimate associations with plants and animals [1,2,3,4]. Given their universal distribution, *Pseudomonas* bacteria have become the focus of numerous aspects of research [5,6]. For example, the soil bacterium *Pseudomonas putida*, a well–established model organism for cloning and gene expression, is able to host harsh biochemical reactions and has been engineered as a synthetic biology chassis for industrial production of biofuels and fine chemicals [7]. *Pseudomonas syringae* is a common foliar bacterium responsible for many important plant diseases. Genome–wide mutant screening has revealed the detailed mechanisms of *P. syringae* pathogenesis, for example, the regulation of the type III secretion system [8]. Despite much of studies depending on the sophisticated genetic work, however, often native isolates are difficult to genetically manipulate, and most molecular tools are applicable only to certain types of mutations or *Pseudomonas* species. Therefore, the development of new efficient genetic manipulation methods remains open in such a diverse genus.

*Pseudomonas fulva* is a novel species within the *P. putida* group and has been isolated from plants (such as banana, rice, sugar cane, and maize), hospitals, and other environments, which play many different roles under varying conditions. For instance, *P. fulva* strains isolated from contaminated soil and active sludge actively degrade environmental contaminants, thereby exhibiting great potential in bioremediation [9,10,11]. Furthermore, the rhizobacterium *P. fulva* PS9.1, by colonizing the surface of their host plant (maize) can improve plant growth and suppress the growth of phytopathogens [12]. Interestingly, *P. fulva* was also involved in the intimate bacterial–fungal interactions that stimulate the effective production of an anticancer therapy agent (hypocrellins) from bambusicolous *Shiraia* fungi [13]. Furthermore, some *P. fulva* bacteria are also relevant to human health and could be an opportunistic human pathogen that may cause infections, such as urosepsis, bacteremia, and adult bacterial meningitis [14,15,16].

Despite the growing literature revealing diverse environmental adaptations and functionalities, molecular mechanical studies of *P. fulva* are still scarce, partially due to a lack of genetic tools. It is well recognized, however, that the application of efficient and convenient genetic manipulation techniques is essential for the research of the bacterial physiological and metabolic characteristics, and some past efforts have been given to different *Pseudomonas* species, such as *Pseudomonas aeruginosa* [17,18,19,20], *Pseudomonas putida* [21,22,23], *Pseudomonas syringae* pv. actinidiae [24] and *Pseudomonas fluorescens* [25]. However, some procedures are still time–consuming and laborious. For instance, to construct a deletion mutant in *P. aeruginosa*, a two–step selection process is often required. First, a target gene is replaced by an antibiotic marker via homologous recombination. Second, the antibiotic marker is eliminated with the help of the FLP recombinase, leaving a scar sequence in place of the deleted gene [17]. Unfortunately, there is no research on the genetic manipulation of *P. fulva* hitherto.

**Figure 1:**
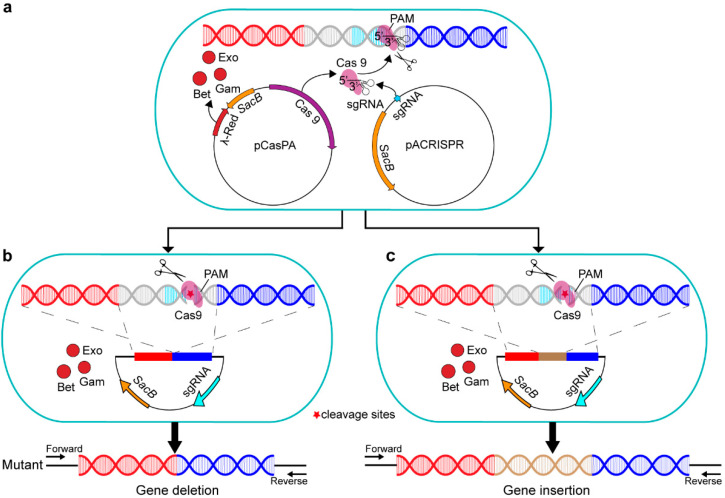
The diagram of the gene–editing approach based on the pCasPA/pACRISPR system in *P. fulva*. (**a**) The CRISPR–Cas9 system recognizes a 5′–NGG–3′ PAM sequence for specific cleavage activity, generating the double–stranded DNA break. (**b**) Homologous recombination–mediated gene deletion and (**c**) gene insertion. Exo, Gam, and Bet represent the phage λ–Red recombination proteins. The asterisk indicates the cleavage site.

Currently, there are three mainstream genome–editing tools, namely zinc finger nucleases (ZFNs) [26], transcription activator–like effector nucleases (TALENs) [27], and the RNA–guided CRISPR (clustered regularly interspaced short palindromic repeats)–Cas (CRISPR–associated) nucleases systems [28]. However, the ZFNs and TALENs systems are difficult to use and are lab– and cost–consuming [29]. Due to the advantages of simple design, low cost, high efficiency, good repeatability, and short cycle, CRISPR–Cas systems are becoming the most widely used genome–editing technology in recent years [30]. CRISPR–Cas technologies have enabled efficient programmable gene editing in a variety of eukaryotes and prokaryotes [31], such as mammalian cells [32], nematodes [33], and *E. coil* [34]. By base–pairing between the 5′end of a single guide RNA (sgRNA) and the target DNA [35], the Cas9 DNA nuclease and sgRNA complex can accurately cleavage the targeted locus in the genome, generating a double–stranded DNA break (DSB) (Figure 1a). The main concern about using CRISPR/Cas9 is the rational sgRNA design, and previous experiments showed that some sgRNAs were less efficient or even inactive [36,37]. Another concern is about the off–targeting effect [38], which may lead to further mutations being introduced in undesired genomic locations [39]. The off–target mutations are commonly due to PAM and sgRNA mismatches. Currently, various bioinformatics tools have been developed to help predict and reduce off–target modifications [40,41,42,43,44]. The DSB can be repaired by either nonhomologous end–joining (NHEJ) [45] or by high–fidelity homology–directed repair (HDR) [46], leading to accurate gene editing by giving the homologous repair template (Figure 1b,c). Due to a lack of the repair pathway of NHEJ, bacterial cells with DSB can survive after homologous recombination.

In this study, we developed a highly efficient and convenient gene–editing method in *P. fulva* with CRISPR–Cas9, based on the HDR and the phage λ–Red recombination system. The phage recombination systems, such as λ–Red and RecET, have shown the preeminent capacity of homologous recombination in a variety of organisms [34,47,48,49]. Here, we investigated the optimal experimental conditions for genetic manipulation of *P. fulva* and performed the precise gene deletion in genes with different lengths (380 bp, 540 bp, 549 bp, 609 bp, 796 bp, and 1486 bp, respectively), which all demonstrated high efficiency (up to 100%). In particular, this method was validated by the deletion of an antibiotic resistance gene, *oqxB* (the deletion of 1486 bp), and we found that the growth of this clean deletion mutant of *P. fulva* slowed down in the LB medium containing the antibiotic chloramphenicol. We also inserted a marker gene, green fluorescent protein *gfp* (the insertion of 978 bp), into the bacterial genome, and an intense fluorescence was detected in fusion constructs. This gene–editing method developed here would greatly simplify the genetic manipulation of *P. fulva* species and accelerate a wide variety of investigations.

## 2. Results and Discussion

### 2.1. Construction of a Two–Plasmid CRISPR–Cas9 System in P. fulva

**Figure 2:**
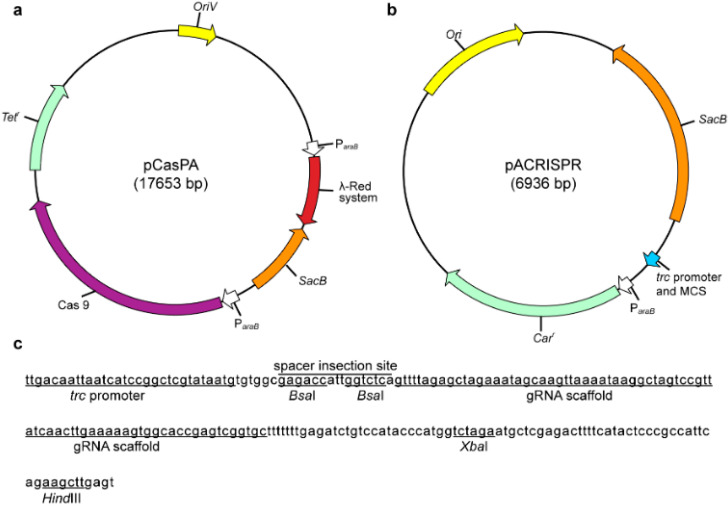
The map of the two–plasmid CRISPR–Cas9 system and multiple cloning sites. (**a**) Genetic and physical map of the plasmid pCasPA. RNA–guided Cas9 protein and the phage λ–Red recombination system were induced under the L–arabinose–inducible *araB* promoter (P*_araB_*). *OriV*, incP origin of replication. *Tet^r^*, the tetracycline–resistance gene of *E. coil* and *P. fulva* for selection. *SacB*, the counter–selectable marker gene used for plasmid curing after genome editing. (**b**) Genetic and physical map of the plasmid pACRISPR. *Ori*, high–copy–number origin of replication. *trc* promoter, the sgRNA expression promoter. MCS, multiple cloning sites. *Car^r^*, the tetracycline–resistance gene of *E. coil* and *P. fulva* for selection. *SacB*, the counter–selectable marker gene used for plasmid curing after genome editing. (**c**) The sequence of the MCS of the pACRISPR. *Bsa*I sites, for the insertion of sgRNA. *Xba*I and *Hind*III sites, for Gibson assembly of the homologous repair arms.

To develop an efficient and convenient genetic manipulation method in *P. fulva*, we sought to harness a two–plasmid system for CRISPR/Cas9–mediated gene editing that has been successfully used in *E. coli* and some other bacteria [49,50]. We first employed the plasmid pCasPA for the expression of the Cas9 nuclease and the λ–Red recombination proteins, including Exo, Gam, and Bet, which are driven by the L–arabinose–inducible promoter P*_araB_* [51] (Figure 2a).

Next, another plasmid pACRISPR (Figure 2b) was used to express the sgRNA which was driven by the well–studied strong promoter *trc* [52]. The pACRISPR plasmid contained two seamless cloning sites, *Bsa*I (Figure 2c), and we used the Golden Gate assembly [53] to insert a 20–nt spacer sequence within the *Bsa*I site; the *Xba*I and *Hind*III sites (Figure 2c) were used for the assembly of homologous repair arms by the Gibson assembly [54]. In addition, the lethal gene *sacB*, conferring sucrose sensitivity, was introduced into the two plasmids as a counter–selectable marker to facilitate rapid plasmid curing after genome editing [55].

### 2.2. Estimation of Transformation Parameters for Higher Transformation Efficiency

Transformation of the two types of plasmids into native isolates often exhibits low efficiency, particularly the plasmid carrying Cas9 nuclease. For instance, the toxicity of Cas9 protein has been observed in various bacterial species. Bacteria also have their own defense system, for example, restriction modification (RM), to prevent foreign DNA from entering their cells [56]. Therefore, it was essential to estimate various transformation parameters for better transformation efficiency in *P. fulva*.

**Figure 3:**
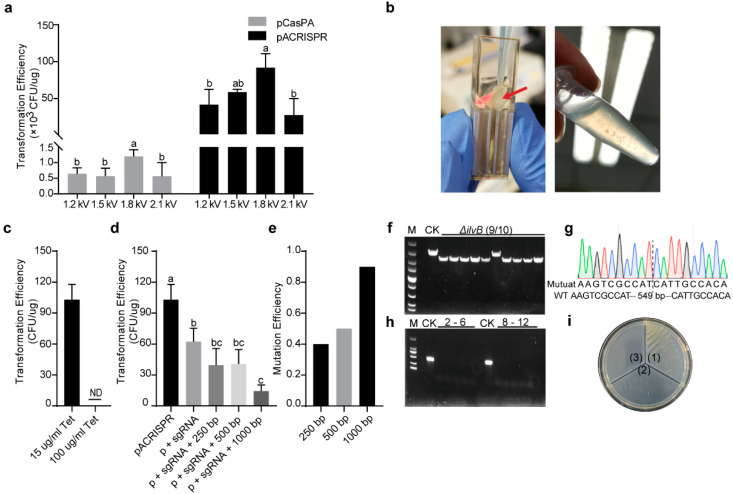
The pCasPA/pACRISPR system–mediated gene editing and plasmid curing after editing in *P. fulva*. (**a**) Effect of different electroporation pulse strengths for transformation efficiency of the two plasmids. (**b**) *P. fulva* cells harboring the pCasPA became flocculent by electroporation (left) and bacteria lysed after recovery (right) when incubated in LB with a high concentration (100 μg/mL) of tetracycline, indicating failure in preparations of *P. fulva* competent cells for the transformation of the second plasmid pACRISPR. The arrow indicates the floccule after electric pulsing. (**c**) The pACRISPR transformation efficiency of *P. fulva* cells harboring the pCasPA incubated under different tetracycline concentrations (15 or 100 μg/mL). ND, not detected (no single colony). (**d**) Transformation efficiency of *P. fulva* harboring both pCasPA and pACRISPR that assembled with sgRNA and different lengths of homologous repair arms. (**e**) Effect of different lengths of homologous repair arms for gene–editing efficiency. (**f**) Agarose gel electrophoresis and (**g**) Sanger sequencing further verified the edition efficiency of the gene *ilvB* (9/10). M, DNA marker (from up to down 5000 bp, 3000 bp, 2000 bp, 1500 bp, 1000 bp, 750 bp, 500 bp, 250 bp and 100 bp). (**h**) There were no positive PCR bands when using the specific primers for detecting genome–editing plasmids after plasmid curing. 2–6, PCR amplification using pCasPA–specific primers; 8–12, PCR amplification using pACRISPR–specific primers. M, DNA marker (from up to down 2000 bp, 1000 bp, 750 bp, 500 bp, 250 bp and 100 bp). (**i**) Plate streaking indicated the success of plasmid curing. (1), LB agar without antibiotic; (2) LB agar containing 100 μg/mL tetracycline; (3) LB agar containing 150 μg/mL carbenicillin. Statistically significant differences are annotated with different letters (*p* < 0.05).

We first tested different pulse strengths (1.2 kV, 1.5 kV, 1.8 kV, and 2.1 kV), and found that both plasmids had the highest transformation efficiency in the pulse strength of 1.8 kV, which reached 1 × 10^3^ and 1 × 10^5^ transformants per μg of plasmid, respectively (Figure 3a). Multiple factors, including the source, size, and concentration of the plasmids, may impact transformation efficiency. For instance, it has been reported that the bigger plasmid usually has lower transformation efficiency [57]. In our study, the two kinds of editing plasmids (pCasPA vs. pACRISPR) displayed a 100–fold difference in efficiency, most probably due to their difference in size (17571 bp vs. 6953 bp).

We next transformed 1 μg of pCasPA plasmids into the *P. fulva* native strain for a single transformation reaction, which yielded approximately 10^3^ colonies. After the induction by L–arabinose for 2 h, the *P. fulva* cells containing the pCasPA plasmid were collected and prepared as the electrocompetent cells for the further pACRISPR transformation. This electroporation was also applied with a pulse strength of 1.8 kV, as described above. However, surprisingly, there was no colony growing on the agar plate, indicating the failure of the pACRISPR transformation. We also found that the competent cell became flocculent after electroporation, and bacteria lysed after 1.5 h of recovery (Figure 3b). We further tested different pulse strengths, however, the results were the same. Considering that the effects of electric fields on bacteria are also related to the physiological properties of the bacterial cells [58], the high concentration of antibiotics (LB broth was added 100 μg/mL tetracycline during incubation) was used to avoid the plasmid loss in cell replication that may trigger adverse effects on bacteria, and further decrease the transformation efficiency. Therefore, we decreased the tetracycline concentration to 15 μg/mL for preparing competent cells. After electroporation, the cells were spread on LB selection plates containing antibiotics, and colonies were successfully recovered compared with the high concentration of antibiotics used (Figure 3c, indicating that *P. fulva* cells had double–antibiotic resistance due to the transformed two plasmids. A previous study reported that prolonged growth (>90 min) will lead to cell lysis [20]; interestingly, in our study, a high concentration of antibiotics used in the preparation of competent cells also caused cell lysis. Therefore, our work may also help to improve competent cell preparation from *Pseudomonas* native isolates.

### 2.3. Analysis of Multigene Editing Efficiencies with the Established Two–Plasmid System in P. fulva

To further assess the performance of the established CRISPR/Cas9–mediated genome editing in *P. fulva*, we performed different genomic modification approaches across a wide range of genes of different lengths. The gene *ilvB* (acetolactate synthase) and the other four genes, *gene0486* (DUF2790 domain–containing protein), *gene4464* (general transcription factor 3C polypeptide), *gabP* (GABA permease), and *thiC* (phosphomethylpyrimidine synthase), were subjected to different genomic modifications. Moreover, the method was validated by deleting the antibiotic resistance gene *oqxB*, as well as by inserting the *gfp* gene.

Firstly, three different kinds of plasmids, including the empty pACRISPR plasmid (as negative control), pACRISPR–*ilvB*–sg (assembly of pACRISPR and only the sgRNA of the target gene), and pACRISPR–*ilvB* (assembly of pACRISPR, the sgRNA of the target gene, and different homologous repair arms) were separately electroporated into the *P. fulva* competent cells harboring the pCasPA plasmid for gene editing, by using the transformation parameters suggested above. The transformation efficiency of the pACRISPR containing the 20–nt *ilvB* spacer was already significantly lower than that of the empty plasmid (Figure 3d). This is probably because the introduction of the *ilvB* spacer produced an intact sgRNA that directed the Cas9 endonuclease to the *ilvB* gene locus to create a double–stranded break, which led to the death of cells [35]. Based on the homologous recombination system driven by the L–arabinose, we further evaluated the gene–editing efficiency of the CRISPR–Cas9 system by employing different lengths of homologous recombination arms. With the extension of the homologous repair arms (from 250 bp to 500 bp to 1000 bp), the larger pACRISPR–*ilvB* plasmids have lower transformation efficiency (Figure 3d). Under different homologous repair arms, the *ilvB* gene in the colony containing the pACRISPR–*ilvB* plasmid was successfully deleted with an efficiency of 4/10, 5/10, and 9/10, as confirmed by both PCR and sequencing (Figure 3e–g). Notably, the longer homologous repair arms significantly increased the gene-editing efficiency, albeit with a decreasing transformation efficiency.

The count-selectable gene *sacB* was employed for curing the plasmids after genome editing. The culture of mutant strain was diluted and plated onto the LB plates with 5% (*w*/*v*) sucrose for 24 h. A single colony from the plate containing sucrose was randomly picked and streaked onto three different types of LB agar plates (no antibiotics, 100 mg/mL tetracycline, and 150 mg/mL carbenicillin, respectively). The PCR revealed no bands with the specific primers after curing (Figure 3h). Meanwhile, it also grew normally on the agar without antibiotics; in contrast, no growth was observed on the other two parts containing 100 mg/mL tetracycline or 150 mg/mL carbenicillin, respectively (Figure 3i).

**Figure 4:**
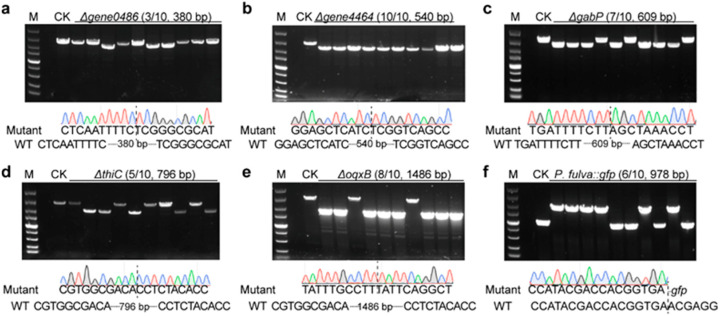
Analysis of multigene editing efficiencies using the developed pCasPA/pACRISPR system in *P. fulva*. The editing efficiency of (**a**) *gene0486* (deletion of 380 bp), (**b**) *gene4464* (deletion of 540 bp), (**c**) *gabP* (deletion of 609 bp), (**d**) *thiC* (deletion of 796 bp), (**e**) *oqxB* (deletion of 1486 bp), and (**f**) *gfp* (insertion of 978 bp) was verified by both agarose gel electrophoresis and Sanger sequencing. M, DNA marker (from up to down 5000 bp, 3000 bp, 2000 bp, 1500 bp, 1000 bp, 750 bp, 500 bp, 250 bp, and 100 bp).

**Figure 5:**
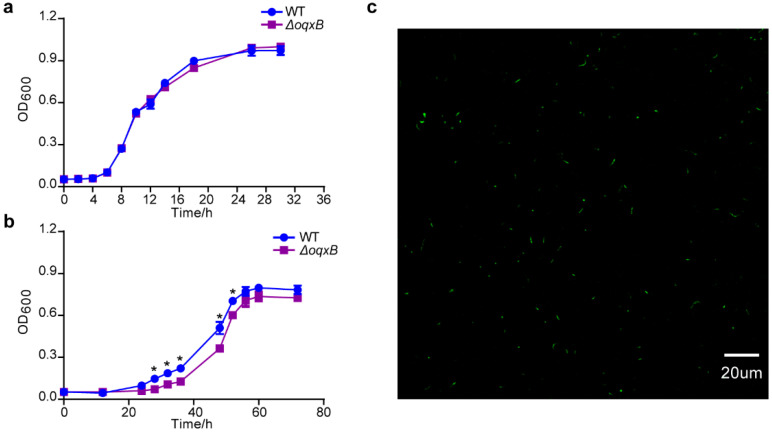
Application of the pCasPA/pACRISPR system–mediated gene editing in the characterization of *P. fulva*. (**a**) No significant differences were observed between wild type and the Δ*oqxB* mutant when grown under normal conditions. (**b**) Compared to the wild type, the growth of the Δ*oqxB* mutant significantly slowed down when cultured in LB containing the antibiotic chloramphenicol. (**c**) The fluorescence of the *P. fulva::gfp* mutant after *gfp* insertion. ∗, representing significant difference (*p* < 0.05).

Due to higher editing efficiency when using the 1000 bp homologous repair arm, we further employed the longer repair templates for deletion of other genes, including *gene4464*, *gene0486*, *gabP*, *thiC*, and *oqxB*; the deletion efficiency of them were 3/10, 10/10, 7/10, 5/10, and 8/10, respectively (Figure 4a–e). In addition, since the gene *oqxB* was predicted to be related to the chloramphenicol resistance by ResFinder [59], we used wild type and the mutant to conduct a drug resistance test. Furthermore, there was no difference between the two strains when cultured in the LB broth without antibiotics (Figure 5a). However, compared to the wild type, the growth of the mutant significantly slowed down when cultured in the LB broth containing 20 μg/mL chloramphenicol (Figure 5b), indicating that the deletion of the resistance gene affected bacterial fitness under stress conditions.

Apart from gene deletion, we further assessed the capacity of the two–plasmid system for gene insertion in *P. fulva*. We inserted the *gfp* gene with *trc* promoter into the ZJU1 genome using this system. The mutant strain was verified by PCR after plasmid curing (Figure 4f), and the green fluorescence was visualized by a fluorescence microscope, too (Figure 5c). Given the easy and efficient genome editing, our system developed here will facilitate future physiological and metabolic analysis in *P. fulva*. Notably, to further investigate functional genes, the protein level validation should follow up gene editing.

## 3. Materials and Methods

### 3.1. Bacteria, Plasmids, Primers, and Growth Conditions

The bacterial strains, plasmids, and primers used in this study are listed in Supplementary Tables S1–S3, respectively. *E. coil* and *P. fulva* ZJU1 native isolate were grown in LB broth at 37 °C and the antibiotics were added when necessary.

### 3.2. Plasmid Construction

The plasmid pCasPA that carried λ–Red system and Cas9 nuclease with the *araB* promoter (*P_araB_*), and the plasmid pACRISPR that expressed sgRNA and homologous recombination repair templates, were a gift from Dr. Quanjiang Ji [60]. To construct the plasmid for genetic editing of *P. fulva*, the optimal 20–nt DNA sequence was designed for the target gene of *P. fulva* via CRISPOR [44]. The phosphorylation product of the oligos was inserted into pACRISPR. Two homology arm fragments (250, 500, or 1000 bp for each other) flanking the target gene were amplified from *P. fulva* genomic DNA, and the fragments were ligated into pACRISPR that was assembled with sgRNA. The detailed method is as follows.

Firstly, the two oligos for the target gene were designed in the following form:

Forward, 5′ GTGGNNNNNNNNNNNNNNNNNNNN 3′

Reverse, 3′ NNNNNNNNNNNNNNNNNNNNCAAA 5′

Then, the phosphorylation of the oligos proceeded with the following method. An amount of 1 μL of T4 polynucleotide kinase (NEB, Ipswich, MA, USA), 1 μL of 10 × T4 polynucleotide kinase buffer (NEB, Ipswich, MA, USA), 5 μL of each sgRNA oligos (100 μM), and 34 μL of ddH_2_O was mixed and phosphorylated at 37 °C for 30 min, and subsequently heated at 95 °C for 3 min, annealed with a gradually decreasing temperature (−0.1 °C per 3 s) to room temperature. The phosphorylation product was diluted up to 100 times before use. Meanwhile, 1 μg pACRISPR was linearized by *Bsa*I–HF (NEB, Ipswich, MA, USA) at 37 °C for 15 min. The linearized product was separated by agarose gel electrophoresis in 1% gel and purified by the E.Z.N.A.^®^ Gel Extraction Kit (OMEGA, Norcross, GA, USA). The construction of pACRISPR ligated with sgRNA was performed with 0.5 μL of T4 DNA Ligase (Takara, Shiga, Japan), 1 μL of 10 × T4 DNA Ligase Buffer (Takara, Shiga, Japan), 20 ng linearized pACRISPR, and 1 μL of annealed sgRNA oligos (100 nM) in a 10 μL reaction mix at 16 °C overnight. The reaction product was introduced in *E. coil* DH5α competent cells (Vazyme, Nanjing, China), and the transformed cells were plated and selected with 50 μg/mL of carbenicillin. To verify the insertion of sgRNA sequences, a PCR reaction was performed with the primers CRISPR–F and CRISPR–R (Table S2). PCR products were sequenced in Sangon Biotech (Shanghai, China) and aligned by MEGA X to confirm the insertion. Next, the pACRISPR assembled with sgRNA was extracted by the E.Z.N.A.^®^ Plasmid Kit (OMEGA, Norcross, GA, USA), and linearized by *Xba*I and *Hind*III as the following steps. An amount of 1 μg of plasmid, 1 μL of QuickCut™ *Xba*I (Takara, Shiga, Japan), 1 μL of QuickCut™ *Hind*III (Takara, Shiga, Japan), and 1 μL of 10 × QuickCut Green Buffer (Takara, Shiga, Japan) were mixed up to 10 μL with ddH_2_O. After the 5 min–incubation, the product was purified as the methods mentioned above. For the assembly of the homologous repair arm, different lengths of the DNA sequence of the upstream and downstream of the target gene were selected, respectively. Donor sequences that typically contain 250 bp upstream and 250 bp downstream (or another length) of the editing sites with a 15 bp overlap of the *Xho*I/*Hind*III–digested pACRISPR plasmid at each end were amplified by PCR (Primer in Table S2). The overlap could be created via PCR with primers that contain a 5′ end that is identical to an adjacent segment and a 3′ end that anneals to the target sequence.

The forward primer of the upstream is in this form:

5′tgtccatacccatggTCTAGANNNNNNNNNNNNNNNNNNNNN 3′

The reverse primer of the downstream is in this form:

5′gggagtatgaaaagtAAGCCTNNNNNNNNNNNNNNNNNNNNNNN 3′

The two DNA fragments were assembled into the digested plasmid using Gibson assembly: 2 × ClonExpress Mix (Vazyme, Nanjing, China), 0.03 pmol *Xba*I/*Hind*III linearized plasmid, 0.03 pmol upstream, and 0.03 pmol downstream were mixed up to 10 μL with ddH_2_O. The reaction solution was incubated at 50 °C for 15 min. For the gene insertion, an external 30 pmol DNA fragment with the overlap was added to the reaction solution. The product was transformed into *E. coli* DH5α competent cells and was selected as described above. The successful construction of the plasmid pACRISPR (ligated sgRNA and homologous repair arm) was verified by PCR and sequencing with the primers CRISPR–F and CRISPR–R (Table S2).

### 3.3. Preparation of Competent Cells

Electrocompetent cells were prepared according to a previously reported method with slight modifications [61,62]. Briefly, *P. fulva* cultured in 1 mL of LB medium overnight was incubated in 100 mL of fresh LB medium as 1:100 diluted at 37 °C. When the optical density at 600 nm (OD_600_) of the culture reached ~0.4 (Spectrophotometer, Thermo Fisher Scientific, Waltham, MA, USA), the cells were chilled on ice for 30 min and then harvested by centrifugation at 6000 rpm for 5 min. The resulting supernatant was discarded, and the pellet was washed twice using sterile ice–cold 10% (*v*/*v*) glycerol. Afterward, the resulting cells were resuspended in 1 mL of glycerol (10% *v*/*v*) and divided into 50 μL aliquots for later use.

A colony of *P. fulva* harboring pCasPA was picked from the plate and cultured in the LB medium at 37 °C overnight. The cells were seeded in 100 mL of LB medium (1:100) and allowed to grow at 37 °C until the OD_600_ ~0.4 with 15 or 100 μg/mL of tetracycline. Meanwhile, L–arabinose was added into the cell culture to give a final concentration of 2 mg/mL for induction and expression of the Cas9 gene and λ–Red system. The cells were prepared for the electrocompetent cells as described above when the OD_600_ reached ~0.5.

### 3.4. Selection of Electrotransformation Parameters

For the selection of electrotransformation parameters, 5 μL of pCasPA or pACRISPR plasmids were mixed with 50 μL of *P. fulva* electrocompetent cells. Then, the electroporation was performed using the MicroPulser (Bio–Rad, Hercules, CA, USA) with a 1 mm ice–cold cuvette (Bio–Rad, Hercules, CA, USA) under the different voltages (1.2 kV, 1.5 kV, 1.8 kV, and 2.1 kV). After electroporation, 1 mL of LB broth (without antibiotic) was added, and the mixture was transferred into a 1.5 mL tube and incubated at 37 °C for 1.5 h. All cells were plated onto the LB plate containing 100 μg/mL of tetracycline (the counter–selectable marker for pCasPA) or 100 μg/mL of carbenicillin (the counter–selectable marker for pACRISPR) and incubated at 37 °C overnight for selection.

A 5 μL pACRISPR of plasmid and 50 μL of pCasPA harboring electrocompetent cells were mixed and transferred to a 1 mm ice–cold cuvette. After electroporation with 1.8 kV and the 1.5 h–resuscitation at 37 °C, the cells were plated onto the LB agar plate containing 100 μg/mL of tetracycline and 150 μg/mL of carbenicillin for selection. The electrotransformation efficiency was calculated in colony–forming units (CFU)/μg of plasmid DNA Transformation efficiency (CFU/μg) = Number of transformants (CFU)/DNA added (μg)

### 3.5. Gene Editing and Plasmid Curing

The constructed plasmid pACRISPR assembled with sgRNA and homologous repair arms was transformed into the *P. fulva* competent cells harboring pCasPA via electrotransformation. The editing efficiency was evaluated by PCR and sequencing (Primers in Table S2). The count–selectable gene *sacB* was applied for plasmids curing in the presence of sucrose after editing. The mutant strain containing both two plasmids was cultured in the fresh LB medium overnight. The culture was diluted and a 100 μL of diluted culture was plated onto the LB plates containing 5% sucrose (*w*/*v*). In some cases, multiple (2 to 3) rounds of streaking on the plates containing sucrose were required for thorough plasmid curing. A single colony was selected, and the curing was verified by the failure of growth in the LB plate with tetracycline or carbenicillin. Meanwhile, colonies were verified by colony PCR using Taq DNA polymerase (Takara, Shiga, Japan) with the indicated primers, CAS–F and CAS–R (Primers in Table S2) [63].

### 3.6. Measurement of the Growth Curve

The single colony of mutant and WT strain were inoculated in 1 mL of LB and incubated overnight at 37 °C, 200 rpm. Then, a 20 μL culture was dropped into 20 mL of LB without or with chloramphenicol, respectively. The growth curve was generated from the data recorded at regular intervals. Each experimental group had three replicates.

### 3.7. Statistical Analysis

All biochemical analysis was performed in triplicate, and the values were expressed as the standard error mean. Differences between groups were compared by one–way ANOVA, followed by LSD. These analyses were performed with the Statistical Package for the Social Sciences, version 20.0 (SPSS v21.0, Chicago, IL, USA). Differences were considered to be significant in all statistical tests with a *p* < 0.05.

## 4. Conclusions

To the best of our knowledge, this is the first application of CRISPR–Cas9 technology on the genome engineering of *P. fulva*. Our results showed that this system can modify the genome with single–gene deletion and insertion, and the efficiency of gene editing is up to 100%. Taken together, these approaches significantly improve the ability to manipulate the *P. fulva* genome. This highly efficient and versatile CRISPR–Cas9 system could also be extended to other *Pseudomonas* bacteria found in nature. Together with other studies, the greatly improved procedures for manipulating bacterial genome will help pave the way for the physiological and metabolic characterization of the vast majority of non–model microorganisms.

## Data Availability

Not applicable.

## Supplementary Materials

The following supporting information can be downloaded at: https://www.mdpi.com/article/10.3390/ijms23105443/s1.
