# Supplementary material for: CRISPR/Cas9–Mediated Genome Editing for Pseudomonas fulva, a Novel Pseudomonas Species with Clinical, Animal, and Plant–Associated Isolates
Source: Int J Mol Sci. 2022 May 13;23(10):5443. doi: 10.3390/ijms23105443 (PMC9145825; doi:10.3390/ijms23105443)
Supplement: Supplementary file 1 [file ijms-23-05443-s001.zip › ijms-1700134-supplementary.pdf]

Supplementary Tables

Table S1. Bacterial strains used in this study.

Strains	Description	Reference
E. coil	-	Lab stock
P. fulva ZJU1	wild type	Lab stock
ZJU1- pCasPA	ZJU1 harboring pCasPA	This study
ZJU1- pACRISPR	ZJU1 harboring pACRISPR	This study
ZJU1 Δ *ilvB*	ZJU1 *ilvB* gene deleted with 1000 + 1000 bp homologous repair arms	This study
ZJU1 Δ *gene0486*	ZJU1 *gene0486* gene deleted	This study
ZJU1 Δ *gene4464*	ZJU1 *gene4464* gene deleted	This study
ZJU1 Δ *thiC*	ZJU1 *thiC* gene deleted	This study
ZJU1 Δ *gabP*	ZJU1 *gabP* gene deleted	This study
ZJU1 Δ *oqxB*	ZJU1 *oqxB* gene deleted	This study
ZJU1:: *gfp*	ZJU1 *gfp* gene inserted	This study

Table S2. Primers used in this study.

Primer	Sequence 5'-3'	Description
CRISPR-F	gcgatttacttttcgacctca	verify the insertion of sgRNA and repair template
CRISPR-R	tacgccaagctatttaggtg	
CAS-F	cgtatctaaatgccgtcgttg	specific primers of pCasPA
CAS-R	ctgaataagctaccgttggac	
ilvB-sgRNA-F	agcaaacgcatatttggcgtcgg	sgRNA sequence for deletion of *ilvB*
ilvB- sgRNA -R	ccgacgccaaatatgcgtttgct	
gene0486-sgRNA-F	agccgatatccagaacaaaccgg	sgRNA sequence for deletion of *gene0486*
gene0486-sgRNA-R	ccggtttgttctggatatcggct	
Gene4464-sgRNA-F	tctcggccttgccaccggtgcgg	sgRNA sequence for deletion of *gene4464*
Gene4464-sgRNA-R	ccgcaccggtggcaaggccgaga	
gabP-sgRNA-F	gtaaacgggccatcgctgaaggg	sgRNA sequence for deletion of *gabP*
gabP-sgRNA-R	cccttcagcgatggcccgtttac	
thiC-sgRNA-F	aagttgcggccgatgatcatcgg	sgRNA sequence for deletion of *thiC*
thiC-sgRNA-R	ccgatgatcatcggccgcaactt	
oqxB-sgRNA-F	gctccagctctcgtattgagcgg	sgRNA sequence for deletion of *oqxB*
oqxB-sgRNA-R	ccgctcaatacgagagctggagc	
gfp-sgRNA-F	ccatacgaccacggtgaacgagg	sgRNA sequence for insertion of *gfp*
gfp-sgRNA-R	cctcgttcaccgtggtcgtatgg	
ilvB-up-F	tgtccatacccatggtctagaCAGGCCAAGGGTACGGTTG	amplification for 1kb upstream repair template
ilvB-up-R	tgtggcaatgATGGCGACTTCCTGATGAACC	
ilvB-down-F	aagtcgccatCATTGCCACACAGAGTTCCAGTC	amplification for 1kb downstream repair template
ilvB-down-R	cactatagaatactcaagcttTATCACTCGATCGGCCACGA	

ilvB-up-F-250<br>*ilvB*-up-R-250	tgtccatacccatggtctagaGGTTCCAACTCTTCGTTGCTGT<br>tgtggcaatgATGGCGACTTCCTGATGAACC	amplification for 250bp upstream repair template
ilvB-down-F-250<br>*ilvB*-down-R-250	aagtcgccatCATTGCCACACAGAGTTCCAGTC<br>cactatagaatactcaagcttCCGGTCGGCAAAGTCTACC	amplification for 250bp downstream repair template
ilvB-up-F-500<br>*ilvB*-up-R-500	tgtccatacccatggtctagaGCTCCTTCCAAACGGGCTT<br>tgtggcaatgATGGCGACTTCCTGATGAACC	amplification for 500bp upstream repair template
ilvB-down-F-500<br>*ilvB*-down-R-500	aagtcgccatCATTGCCACACAGAGTTCCAGTC<br>cactatagaatactcaagcttCACCCACATGCGTGGCCA	amplification for 500bp downstream repair template
gene0486-up-F<br>*gene0486*-up-R	tgtccatacccatggtctagaCGCCCTGGCGCTGGGCCT<br>catgcgcccgaGAAAATTGATATCTGTTAGCTAGCTAACTAGT	amplification for upstream repair template
gene0486-down-F<br>*gene0486*-down-R	tcaattttcTCGGGCGCATGCTGCACG<br>cactatagaatactcaagcttCGGTGCACAAGGGCAGCC	amplification for downstream repair template
Gene4464-up-F<br>*Gene4464*-up-R	tgtccatacccatggtctagaGCATGCGCCGGATGGATG<br>tttgtttcagggctgaccgaGATGAGCTCCTTGGGAAGAAGTAA	amplification for upstream repair template
Gene4464-down-F<br>*Gene4464*-down-R	TCGGTCAGCCCTGAAACAAA<br>cactatagaatactcaagcttTGCAGACTGATTCCACGGCC	amplification for downstream repair template
gabP-up-F<br>*gabP*-up-R	tgtccatacccatggtctagaATTCGCTATTTCAGGAATGCTTC<br>gtggctatgcaggtttagctTAGAAAATCAGCATCCGCCATA	amplification for upstream repair template
gabP-down-F<br>*gabP*-down-R	AGCTAAACCTGCATAGCCACAAA<br>cactatagaatactcaagcttAAGTAGCAGGACTCCAAGCTCACC	amplification for downstream repair template
thiC-up-F<br>*thiC*-up-R	tgtccatacccatggtctagaGGAACCTGTCCACGATCTTTATG<br>cgacaCCTCTACACCCACTTCGACGA	amplification for upstream repair template
thiC-down-F<br>*thiC*-down-R	aagtgggtgtagaggTGTCGCCACGGTCCTCGA<br>cactatagaatactcaagcttGGCGGCCAGGTCGGCATT	amplification for downstream repair template

oqxB-up-F	tgtccatacccatggtctagaGTAAAGCCGAGGAAGCCGG	amplification for upstream repair template
oqxB-up-R	ctgatcctctaCAGGCTCGTCTGCAGGAGG	
oqxB-down-F	acgagcctgTAGAGGATCAGCGCGATGCC	amplification for downstream repair template
oqxB-down-R	cactatagaatactcaagcttACAACAGCTTCCAGCTGTCGA	
gfp-up-F	tgtccatacccatggtctagaGGTGTGACGAGAGGACACTTGG	amplification for upstream repair template
gfp-up-R	catggTCACCGTGGTCGTATGGAGTT	
gfp-F	atacgaccacggtgaCCATGGCGGCCGCTGTCATGGGCCCTTGACAATTAATCA TCCGGCTCGTATAATGTGTGGAATTGTGAGCGGATAACAATTTCACACA AAAAGAGGAGAAAACTAGATGAGTAAAGGAG	amplification for *gfp* gene
gfp-R	gtcagcctcgtATACTCAAGCTTCTGAATGGCGG	
gfp-down-F	cttgagtatACGAGGCTGACCTGAATGTCG	amplification for downstream repair template
gfp-down-R	gggagtatgaaaagtaagcttTAACTGAGGAGTTAGGCTACGGG	

The underline indicates the restriction sites.

Table S3. Plasmids used in this study.

Plasmids	Description	Reference
pCasPA	*Tet*^{r}, for expression of Cas9 protein and λ-Red recombination system	[1]
pACRISPR	*Car*^{r}, for expression of sgRNA and assembling homologous repair arms	[1]
pTRKH3-ermGFP	for amplification *gfp*	[2]
pACRISPR-*ilvB*-1000 bp	pACRISPR derivative for *ilvB* deletion with 1000 + 1000 homologous repair arms	This study
pACRISPR-*ilvB*-250 bp	pACRISPR derivative for *ilvB* deletion with 250 + 250 homologous repair arms	This study
pACRISPR-*ilvB*-500 bp	pACRISPR derivative for *ilvB* deletion with 500 + 500 homologous repair arms	This study
pACRISPR-*gene0486*	pACRISPR derivative for *gene0486* deletion	This study
pACRISPR-*gene4464*	pACRISPR derivative for *gene4464* deletion	This study
pACRISPR-*gabP*	pACRISPR derivative for *gabP* deletion	This study
pACRISPR-*thiC*	pACRISPR derivative for *thiC* deletion	This study
pACRISPR-*oqxB*	pACRISPR derivative for *oqxB* deletion	This study
pACRISPR-*gfp*	pACRISPR derivative for *gfp* insertion	This study

Tet^{r}, tetracycline resistant; *Car*^{r}, carbenicillin resistant;

References:

1. Chen, W.; Zhang, Y.; Zhang, Y.; Pi, Y.; Gu, T.; Song, L.; Wang, Y.; Ji, Q. CRISPR/Cas9-based Genome Editing in Pseudomonas aeruginosa and Cytidine Deaminase-Mediated Base Editing in Pseudomonas Species. *iScience* **2018**, *6*, 222-231, doi:10.1016/j.isci.2018.07.024.
2. Teh, B.S.; Apel, J.; Shao, Y.; Boland, W. Colonization of the Intestinal Tract of the Polyphagous Pest Spodoptera littoralis with the GFP-Tagged Indigenous Gut Bacterium Enterococcus mundtii. *Front. Microbiol.* **2016**, *7*, 928, doi:10.3389/fmicb.2016.00928.
